# Functionalized Polymeric Microneedles for Transdermal Delivery of Ovalbumin Protein Antigen

**DOI:** 10.3390/pharmaceutics17060737

**Published:** 2025-06-04

**Authors:** Yi Liu, Feng Tan, Decheng Zhao, Liwen Zhang, Nianni Zhang, Chengwei Bai, Ziyang Guo, Xiongjian Guan, Guanyu Chen

**Affiliations:** School of Pharmaceutical Sciences (Shenzhen), Sun Yat-sen University Shenzhen Campus, Shenzhen 518107, China

**Keywords:** dissolvable microneedles, transdermal drug delivery, immunomodulation, reactive oxygen species (ROS) regulation, β-glucan, fucoidan, hyaluronic acid, lymphatic targeting, protein antigen delivery

## Abstract

**Background/Objectives:** Microneedles represent an innovative transdermal drug delivery approach, especially for protein antigens. This study aimed to develop a dual-functional, dissolvable microneedle system loaded with β-glucan and fucoidan in a hyaluronic acid matrix to achieve transdermal immunomodulation and reactive oxygen species (ROS) regulation, exploring its potential in inflammatory disease management and antigen delivery. **Methods:** The microneedles were fabricated using a two-step casting method. Their morphology, mechanical strength, and dissolution kinetics were characterized. In vitro experiments evaluated the ROS-modulating effects on human dermal fibroblasts, while in vivo studies on C57 mice investigated immune activation and lymph node accumulation of ovalbumin antigen. **Results:** The microneedles exhibited a mechanical strength exceeding 7.45 N/needle and dissolved within 50 s. β-glucan transiently reduced ROS levels at 6 h followed by a rebound, whereas fucoidan sustained ROS suppression after 12 h. In mice, β-glucan-loaded microneedles triggered local immune activation, and fucoidan-incorporated microneedles enhanced ovalbumin accumulation in lymph nodes by 2.1-fold compared to controls. **Conclusions:** Integrating β-glucan’s immunostimulatory and fucoidan’s ROS-scavenging/lymphatic-targeting properties within a single microneedle platform offers a promising multifunctional strategy for treating inflammatory diseases and delivering protein antigens.

## 1. Introduction

Traditional vaccine delivery methods often cause pain and discomfort due to intramuscular injection. Microneedle (MN) systems have emerged as a revolutionary approach in transdermal drug delivery, offering a safe, minimally invasive, and patient-friendly alternative for protein antigen delivery [1]. These systems enable precise delivery of vaccine components and adjuvants to the target depth within the skin, facilitating robust interactions with cutaneous antigen-presenting cells (APCs) and eliciting potent immune responses [2]. Compared to conventional intramuscular injections, MNs provide significant advantages, including pain-free administration, improved patient compliance, and enhanced immunogenicity due to their “mechanical adjuvant” effect [3]. Recent advancements in tumor vaccine MN have demonstrated their clinical potential: for instance, Chunli Yang et al. developed an ice MN-encapsulating living tumor cell vaccine (TCV) for melanoma immunotherapy [4], while Chang H et al. successfully constructed a DC vaccine and anti-PD-1 antibody co-delivery system using cryomicroneedles [5]. Although current research primarily focuses on skin cancer models, MNs have also shown promise in treating squamous cell carcinoma, cervical cancer, and breast cancer [6]. Moreover, prophylactic tumor models have revealed the remarkable tumor-suppressive capabilities of vaccine-loaded MNs [7,8]. However, challenges remain in expanding their application to broader tumor types, bridging the gap between preclinical studies and clinical applications, and optimizing manufacturing processes and formulation designs [9].

Beta-glucan, hyaluronic acid (HA), and fucoidan are natural and biodegradable materials that exhibit excellent biocompatibility with minimal risk of foreign body residue [10,11]. β-glucan, derived from oats and yeast, activates T cells, macrophages, and natural killer cells, promoting immune cell differentiation and complementing pathway activation [12]. HA, naturally present in human tissues, supports skin hydration and repair while enhancing antigen delivery [13]. Together, these materials create a synergistic effect, improving both the mechanical properties and immunostimulatory potential of the MN system. In addition, utilizing β-glucan/HA to fabricate the MN proposed in this study offers several innovative advantages: (1) material superiority, as β-glucan exhibits immunomodulatory effects by enhancing APC activity and promoting Th1-type immune responses [14], while low-molecular-weight HA targets CD44 receptors to improve antigen uptake and presentation [15]; and (2) technical advantages, as MNs reduce drug dosage, minimize systemic toxicity, and mechanically stimulate the local immune microenvironment [14].

This study aims to develop a novel soluble composite MN based on β-glucan, fucoidan, and HA, designed to establish a transdermal delivery platform with synergistic immunostimulatory effects. We will systematically characterize its critical quality attributes, including mechanical properties (rigidity, puncture force), dissolution kinetics, and biocompatibility [15]. Through in vitro and in vivo studies employing flow cytometry, immunofluorescence staining, and quantitative PCR (qPCR), we will evaluate its safety profile and immunostimulatory efficacy. Notably, β-glucan serves as a natural ligand for the pattern recognition receptor Dectin-1 [16], while HA exhibits CD44-targeting properties [17]; their combination is expected to generate synergistic immune activation. Mechanistic insights into the immunostimulatory effects of the β-glucan/HA complex will be elucidated by analyzing reactive oxygen species (ROS) levels and immune activation markers post-local administration, using flow cytometry coupled with qPCR [18]. The study further establishes a bifunctional synergistic system through co-incorporation of β-glucan and fucoidan: β-Glucan drives directional migration of XCR1^+^ dendritic cells via activation of the IL-1β/CXCL10 signaling axis, while fucoidan optimizes immune-response microenvironments by enhancing antigen lymphatic targeting efficiency. Our goal is to engineer a microneedle system with superior biocompatibility, optimal mechanical strength, high delivery efficiency, and robust immune activation capabilities, thereby providing a scientific foundation for transdermal protein antigen delivery.

## 2. Materials

### 2.1. Reagents and Materials

β-Glucan (purity ≥ 98%) and Rhodamine B were purchased from Shanghai Macklin Biochemical Co., Ltd. (Macklin, Shanghai, China). Hyaluronic acid (molecular weight: 10 kDa) was provided by Bloomage Biotech Co., Ltd. (Bloomage, Jinan, China). N-Acetyl-L-cysteine (NAC) was obtained from Milibor Biotechnology Co., Ltd. (Milibor, Darmstadt, Germany). Isoflurane was supplied by RWD Life Science Co., Ltd. (RWD, Shenzhen, China). The SYLGARD^TM^ 184 Silicone Elastomer Kit was acquired from Dow Corning Corporation (Midland, MI, USA). Polyvinylpyrrolidone (PVP K90) was purchased from Aladdin Biochemical Technology Co., Ltd. (Aladdin, Shanghai, China). Fresh porcine skin was sourced from local markets. A total of 4% paraformaldehyde (PFA) fixative solution was procured from Servicebio Technology Co., Ltd. (Servicebio, Wuhan, China). All other chemicals used were of reagent grade. Ultrapure water was prepared using a Millipak^®^ Express 40 filtration system (Millipore, Burlington, MA, USA; 0.22 µm pore size).

### 2.2. Cell Culture and Biological Reagents

Human dermal fibroblasts (HDFs) were provided by Aunion Biotechnology Co., Ltd. (Aunion, Shanghai, China). Dulbecco’s Modified Eagle Medium (DMEM) was purchased from Gibco^TM^ (Thermo Fisher Scientific, Grand Island, NY, USA). Fetal bovine serum (FBS) was acquired from HyClone^TM^ (Cytiva, Logan, UT, USA). Phosphate-buffered saline (PBS, pH 7.4) was supplied by ACMEC Biochemical Co., Ltd. (ACMEC, Shanghai, China). The Reactive Oxygen Species (ROS) Assay Kit (Cat. No. S0033S) was obtained from Beyotime Biotechnology Institute (Beyotime, Beijing, China). The FastPure Cell/Tissue Total RNA Isolation Kit V2 was purchased from Vazyme Biotech Co., Ltd. (Vazyme, Nanjing, China). Hieff UNICON^®^ qPCR SYBR Green Master Mix was procured from Yeasen Biotechnology Co., Ltd. (Yeasen, Shanghai, China).

## 3. Methods

### 3.1. Mold Preparation

Copper-based MN array molds were used for the preparation of PDMS (polydimethylsiloxane) templates. The basic components and curing agents of the Dow Corning SYLGARD 184 silicone rubber kit were mixed in a 10:1 (*w*/*w*) ratio to a total of 22 g in a beaker. The mixture was placed in a vacuum pump, evacuated to 0.01 mPa, and maintained for 30 min to remove air bubbles. The vacuumed PDMS mixture was poured into clean copper MN array molds while ensuring no air bubbles were introduced. The filled molds were placed back into the vacuum pump to remove any remaining bubbles and then dried overnight in an oven.

### 3.2. β-Glucan and HA Composite Soluble MN Preparation

Using the aforementioned mold, MNs were prepared by casting. A solution containing 0.001‰ *w*/*w* β-glucan and 25% *w*/*w* HA was cast into the MN mold (300 μL per mold). The solution was then centrifuged at 2000 rpm for 30 min to allow it to fill the MN pores, followed by drying at room temperature for 3 h. A 20% *w*/*w* PVP K90 solution (without drug) was added to the dried MN molds as a backing material, which was then centrifuged at 4000 rpm for 6 min and dried for 18 h at room temperature [19,20].

### 3.3. Characterization of Soluble MNs

The MNs were characterized for their morphology, strength, and insertion capabilities. Optical microscopy was used to observe their morphology. Mechanical strength was evaluated using a texture analyzer (Brookfield, Toronto, ON, Canada) to perform compression force testing under the following conditions: target compression, 0.7 mm; wait time, 0 s; trigger load, 0.07 N; test speed, 0.10 mm/s; return speed, 1 mm/s [21].

To further assess the insertion and dissolution performance, 0.005% *w*/*w* rhodamine B was added to the MN formulation. Fresh pig skin was obtained from the butcher and was freshly prepared by being dissected into pieces (3–5 cm square) and cleaned with physiological saline, after which the surface was dried with filter paper. At room temperature (25 °C), the rhodamine B-loaded MNs were inserted into the pig skin with constant force to observe the dye diffusion and MN dissolution. The microneedles would be hydrated and dissolved upon contact with the moisture within the skin.

### 3.4. Effects of β-Glucan and Fucoidan on ROS Production in HDF Cells

HDF cells were cultured in DMEM medium supplemented with 10% fetal bovine serum and 0.1% penicillin-streptomycin. Cells in the logarithmic growth phase with good viability were trypsinized, counted, and adjusted to a density of 5 × 10^5^ cells/mL. A 1 mL aliquot of the cell suspension was added to each well of a 24-well plate and incubated at 37 °C in a 5% CO_2_ humidified incubator. At 0 h, 12 h, and 18 h post-seeding, 50 μL of 2 mg/mL [22,23] β-glucan or fucoidan medium solution was added to the respective wells. N-acetylcysteine (NAC) [24], used as a negative control, was administered at the same time points by adding 10 μL of 500 mM NAC to the control group.

After 24 h of culture, the supernatant was aspirated, and cells were detached using 0.5% trypsin. The cell suspension was transferred to a 1.5 mL centrifuge tube and centrifuged at 3500 rpm for 5 min. The supernatant was discarded, and 50 μL of PBS containing 0.05 μL ROS fluorescent probe was added to each tube, followed by vortex mixing. Cells were stained at 37 °C for 20 min, with vortexing every 5 min. The probe was removed by washing three times with PBS. The mean fluorescence intensity (MFI) of cells in the FITC channel was quantified using flow cytometry, and data were analyzed with GraphPad Prism10 software.

### 3.5. Local Application of Soluble MN

Female C57BL/6 mice were purchased from Zhuhai Bestop (Zhuhai, China) and maintained under controlled conditions (25 ± 1 °C and 50 ± 10% humidity) with free access to food and water. After anesthetizing the mice with isoflurane, the skin hair of mice was shaved and MNs were gently pressed to a designated skin region of the back of the mice for 5 min to allow complete dissolution of needles, followed by observation of the skin condition.

### 3.6. Quantitative Polymerase Chain Reaction (q-PCR) Analysis

qPCR was performed to detect cytokine expression levels in the skin at different time points following MN administration. Mice were shaved and depilated, and the skin treated with MN was harvested for RNA extraction. Reverse transcription was performed to synthesize cDNA, and qPCR was conducted with specific primers for IL-6, IL-12, CXCL10, IL-1β, and β-actin (Table 1).

### 3.7. Laser Confocal Microscopy Observation

The distribution of immune cells in mouse skin following MN treatment was observed using immunofluorescence staining combined with laser confocal microscopy. Tissue sections were stained with antibodies against CD11c and XCR1. XCR1-positive immune cells showed migration, indicating activation of the immune response.

### 3.8. Preparation of Drug-Loaded Microneedles (MNs) and Evaluation of Lymph Node Accumulation of Antigen Delivered via MNs

The fabrication of MNs followed a protocol analogous to Section 3.2, utilizing a 25% (*w*/*v*) hyaluronic acid (HA) matrix solution supplemented with 0.25 mg/kg fucoidan, 1 mg/kg β-glucan, and 0.25 mg/kg ovalbumin-Rhodamine B (OVA-RhB) as the antigenic payload. The hair on the right back of the mice was removed, and MN was applied to the hair removal area. After 4 h, the fluorescence intensity in the ipsilateral lymph nodes was measured using a small animal in vivo imaging system [25].

## 4. Results and Discussion

### 4.1. Microneedle Mold

**Figure 1:**
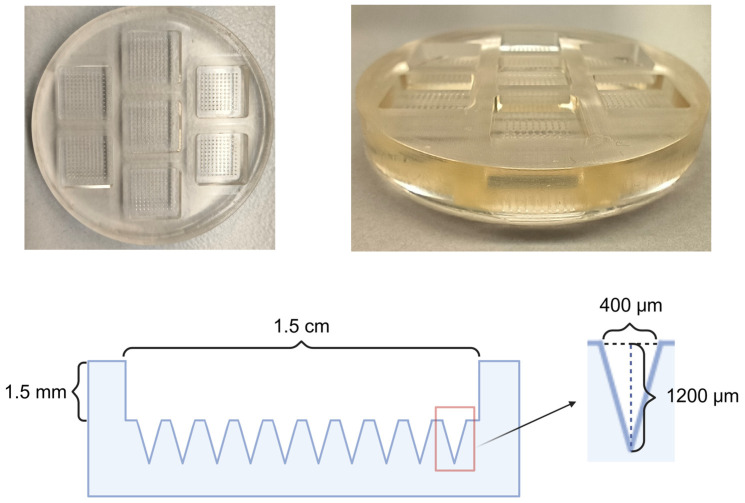
1200 μm MN molds were fabricated. Needle length: 1200 μm (the actual insertion depth does not equal the absolute fabricated needle length). Base diameter: 400 μm. Center-to-center spacing: 1 mm (inter-needle pitch). Patch dimensions: 1.5 cm × 1.5 cm (base area). MN array configuration: 10 × 10 matrix (total of 100 needles per patch). Base height: 1.5 mm.

As illustrated in Figure 1, we successfully fabricated a polydimethylsiloxane (PDMS) negative mold capable of concurrently producing seven microneedle (MN) patches, demonstrating high efficiency, operational convenience, and cost-effectiveness. The MNs prepared using this mold exhibit the following specifications:

### 4.2. Microneedles Characterization

**Figure 2:**
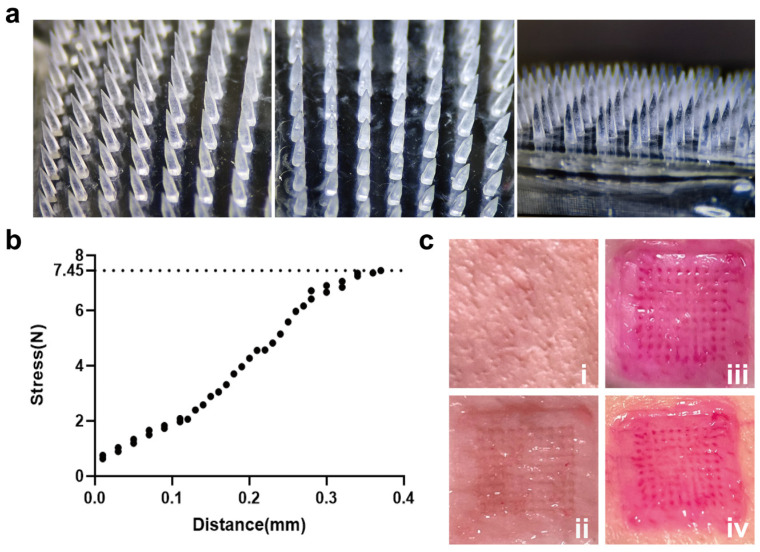
(**a**) MN fabricated from a blend solution of β-glucan and 25% (*w*/*v*) HA; (**b**) load-displacement curve of MN; (**c**) insertion status of MN in porcine skin at different time points. (i. Fresh porcine ex vivo skin; ii. Microneedles form microchannels in porcine skin; iii. Rhodamine B microneedles are inserted into porcine skin; iv. The microneedles are completely dissolved within 5 min and generate microchannels).

The MN in Figure 2a, prepared using the two-step method, showed uniform array spacing and no visible bubbles, precipitation, or stratification. Mechanical characterization using a texture analyzer revealed sufficient strength for transdermal applications, with a maximum load of 7.45 N per needle (Figure 2b), which exceeds the minimum threshold for skin insertion (0.38 N). Furthermore, insertion tests in fresh porcine skin (Figure 2c) confirmed successful epidermal penetration, with the complete dissolution of MN within 5 min post-insertion and visible micropore formation confirmed by Rhodamine B staining.

### 4.3. Effect of β-Glucan and Fucoidan on ROS Production in HDF Cells

**Figure 3:**
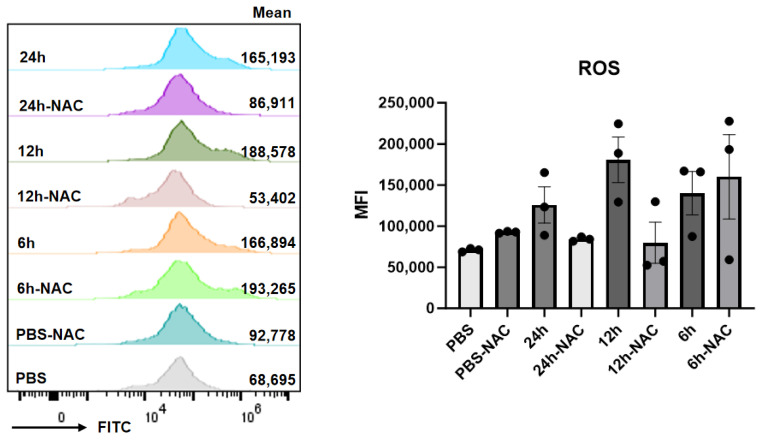
Effect of β-glucan on ROS levels in HDF cells.

The results depicted in Figure 3 demonstrate that treatment with 100 μg/mL β-glucan induced a biphasic modulation of reactive oxygen species (ROS) levels: ROS levels initially increased within the first 6 h, followed by a subsequent decline. This pattern suggests that β-glucan exerts a time-dependent antioxidant effect, albeit with a diminishing response observed over the 12-h experimental duration.

**Figure 4:**
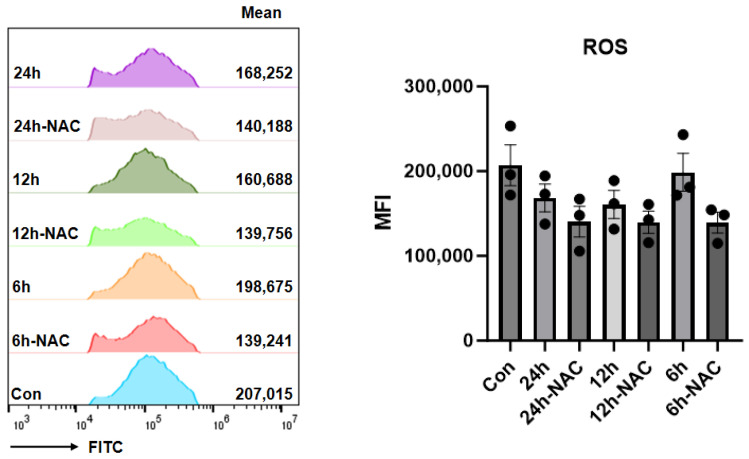
Effect of fucoidan on ROS levels in HDF cells.

In contrast, as illustrated in Figure 4, fucoidan at the same concentration (100 μg/mL) elicited a sustained reduction in ROS levels throughout the 12-h treatment period, maintaining its inhibitory efficacy without attenuation.

### 4.4. Quantitative Polymerase Chain Reaction (q-PCR) Analysis

**Figure 5:**
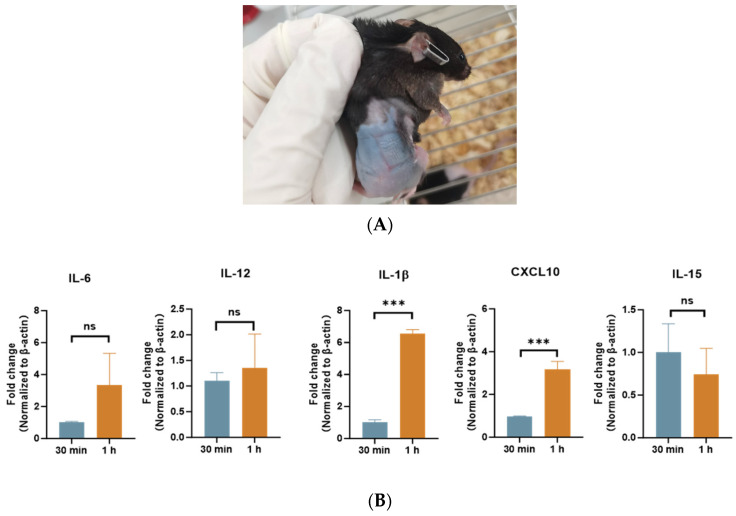
(**A**) Dermal condition of mice following MN administration. (**B**) Expression levels of cytokines in murine skin cells following MN administration. The data were represented as mean ± SD and analyzed by Student’s *t* test. *** *p* < 0.001. n.s. not significant.

Prior to assessing immune responses, histological evaluation (Figure 5A) confirmed the preserved histopathological integrity of murine skin post-MN application, with no evidence of persistent edema, necrosis, or stratum corneum disruption—critical safety prerequisites for subsequent immunological analyses. As shown in Figure 5B, this minimally invasive delivery approach nevertheless induced significant upregulation of IL-1β (1.9-fold, *p* = 0.013) and CXCL10 (2.8-fold, *p* < 0.01) expression levels compared to intact controls, indicating spatially constrained inflammatory activation.

### 4.5. Laser Confocal Microscopy Observation

**Figure 6:**
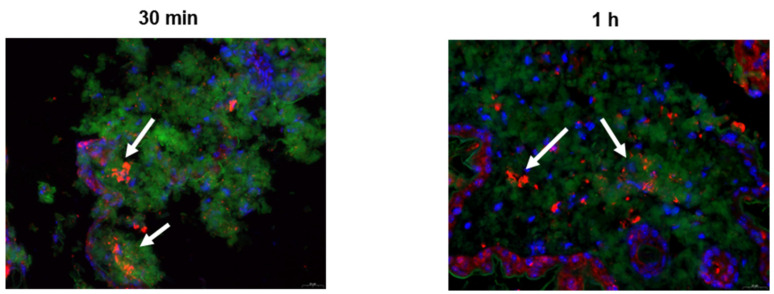
Laser scanning confocal microscopy observation of cytokine expression in murine skin cells post-administration. The arrow indicates DC1 cells for colocalization.

MN treatment resulted in the migration of XCR1-positive immune cells, as visualized by laser confocal microscopy (Figure 6). These cells moved toward the antigen-processing sites, contributing to the initiation of adaptive immune responses.

### 4.6. Lymph Node Accumulation of Protein Delivered via MN

**Figure 7:**
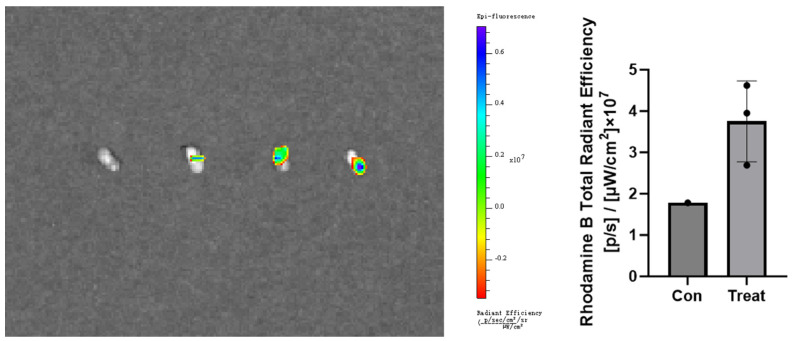
Comparison chart of fluorescence intensity of lymph nodes between the control group and the experimental group.

As shown in Figure 7, compared to the control group treated with blank MN (first panel on the left), the drug-loaded MN exhibited significantly stronger fluorescence signals in the ipsilateral lymph nodes (the panel on the right) 4 h after administration, demonstrating that the MN-delivered drugs were effectively transported to the lymph nodes.

## 5. Conclusions

The β-glucan/fucoidan/HA composite MNs represent a significant advancement in transdermal delivery systems, combining immunomodulatory and antioxidant functionalities. This study demonstrated that the HA matrix ensured mechanical integrity (1200 μm penetration depth, 7.45 N/needle strength), addressing the prior limitations of brittle MN materials. Additionally, it was found that β-glucan elicited a biphasic ROS response in HDFs (initial reduction at 6 h followed by rebound), while fucoidan provided sustained ROS suppression, highlighting their complementary roles in oxidative stress management. Moreover, β-glucan drove localized immune activation (IL-1β, CXCL10 upregulation; DC migration), whereas fucoidan enhanced lymphatic delivery efficiency, as evidenced by a 2.1-fold higher lymph node fluorescence versus blank MNs. This dual-component system offers a paradigm shift from single-agent MN designs, enabling simultaneous immunostimulation and oxidative stress mitigation. Future work should explore the dose optimization of β-glucan/fucoidan ratios, preclinical efficacy in chronic inflammation models, and scalable manufacturing processes. The platform’s versatility positions it for applications ranging from cancer immunotherapy to chronic wound healing.

## Figures and Tables

**Table 1:** qPCR was conducted with specific primers for cytokine expression.

	Forward Primer	Reverse Primer
IL-6	CTCTGCAAGAGACTTCCATCCAGT	GAAGTAGGGAAGGCCGTGG
IL-12	TCTTAGCCAGTCCCGAAACC	TTGGTCCCGTGTGATGTCTTC
CXCL10	GCCGTCATTTTCTGCCTCA	CGTCCTTGCGAGAGGGATC
IL-1β	ATGGCAGAAGTACCTGAGCTCGC	TCAGACAGCCCAGGTCAAAGG
β-actin	GTGGCATCCATGAAACTACAT	GGCATAGAGGTCTTTACGG

## Data Availability

Data is unavailable due to privacy.

## References

[1] Prausnitz M.R., Langer R. (2008). Transdermal drug delivery. Nat. Biotechnol..

[2] Kim Y.C., Park J.H., Prausnitz M.R. (2012). Microneedles for drug and vaccine delivery. Adv. Drug Deliv. Rev..

[3] Lim S.H., Kathuria H., Amir M.H.B., Zhang X., Duong H.T.T., Ho P.C., Kang L. (2021). High resolution photopolymer for 3D printing of personalised microneedle for transdermal delivery of anti-wrinkle small peptide. J. Control. Release.

[4] Yang C., Zhao W., Zhang L., He L., Wang S., Wang J., Xiang M., Yuan X., Gou M. (2025). Intradermal Delivery of Cell Vaccine via Ice Microneedles for Cancer Treatment. Adv. Healthc. Mater..

[5] Chang H., Wen X., Li Z., Ling Z., Zheng Y., Xu C. (2023). Co-delivery of dendritic cell vaccine and anti-PD-1 antibody with cryomicroneedles for combinational immunotherapy. Bioeng. Transl. Med..

[6] Dahri M., Beheshtizadeh N., Seyedpour N., Nakhostin-Ansari A., Aghajani F., Seyedpour S., Masjedi M., Farjadian F., Maleki R., Adibkia K. (2023). Biomaterial-based delivery platforms for transdermal immunotherapy. Biomed. Pharmacother..

[7] Cole G., Ali A.A., McErlean E., Mulholland E.J., Short A., McCrudden C.M., McCaffrey J., Robson T., Kett V.L., Coulter J.A. (2019). DNA vaccination via RALA nanoparticles in a microneedle delivery system induces a potent immune response against the endogenous prostate cancer stem cell antigen. Acta Biomater..

[8] D’Amico C., Fusciello M., Hamdan F., D’Alessio F., Bottega P., Saklauskaite M., Russo S., Cerioni J., Elbadri K., Kemell M. (2025). Transdermal delivery of PeptiCRAd cancer vaccine using microneedle patches. Bioact. Mater..

[9] Edwards C., Shah S.A., Gebhardt T., Jewell C.M. (2023). Exploiting Unique Features of Microneedles to Modulate Immunity. Adv. Mater..

[10] Sousa P., Tavares-Valente D., Amorim M., Azevedo-Silva J., Pintado M., Fernandes J. (2023). β-Glucan extracts as high-value multifunctional ingredients for skin health: A review. Carbohydr. Polym..

[11] Silva A.C.Q., Silvestre A.J.D., Vilela C., Freire C.S.R. (2021). Natural Polymers-Based Materials: A Contribution to a Greener Future. Molecules.

[12] Chan G.C., Chan W.K., Sze D.M. (2009). The effects of beta-glucan on human immune and cancer cells. J. Hematol. Oncol..

[13] Fallacara A., Baldini E., Manfredini S., Vertuani S. (2018). Hyaluronic Acid in the Third Millennium. Polymers.

[14] Zhu D., Yan H., Zhou Y., Nack L.M., Liu J., Parak W.J. (2023). Design of disintegrable nanoassemblies to release multiple small-sized nanoparticles. Adv. Drug Deliv. Rev..

[15] Han Y., Qin X., Lin W., Wang C., Yin X., Wu J., Chen Y., Chen X., Chen T. (2025). Microneedle-Based Approaches for Skin Disease Treatment. Nano-Micro Lett..

[16] Keating S.T., Groh L., van der Heijden C., Rodriguez H., Dos Santos J.C., Fanucchi S., Okabe J., Kaipananickal H., van Puffelen J.H., Helder L. (2020). The Set7 Lysine Methyltransferase Regulates Plasticity in Oxidative Phosphorylation Necessary for Trained Immunity Induced by β-Glucan. Cell Rep..

[17] Iqbal S., Andersson S., Nesta E., Pentinmikko N., Kumar A., Kumar Jha S., Borshagovski D., Webb A., Gebert N., Viitala E.W. (2025). Fetal-like reversion in the regenerating intestine is regulated by mesenchymal asporin. Cell Stem Cell.

[18] Sies H., Mailloux R.J., Jakob U. (2024). Fundamentals of redox regulation in biology. Nat. Rev. Mol. Cell Biol..

[19] Lv H., Gao N., Zhou Q., Wang Y., Ling G., Zhang P. (2023). Collagen-Based Dissolving Microneedles with Flexible Pedestals: A Transdermal Delivery System for Both Anti-Aging and Skin Diseases. Adv. Healthc. Mater..

[20] Makadia H.K., Siegel S.J. (2011). Poly Lactic-co-Glycolic Acid (PLGA) as Biodegradable Controlled Drug Delivery Carrier. Polymers.

[21] Bhatnagar S., Bankar N.G., Kulkarni M.V., Venuganti V.V.K. (2019). Dissolvable microneedle patch containing doxorubicin and docetaxel is effective in 4T1 xenografted breast cancer mouse model. Int. J. Pharm..

[22] Abbas M.F., Karim D.K., Kareem H.R., Kamil M.M., Al-Musawi M.H., Asker M.H., Ghanami M., Shahriari-Khalaji M., Sattar M., Mirhaj M. (2025). Fucoidan and its derivatives: From extraction to cutting-edge biomedical applications. Carbohydr. Polym..

[23] Chen L., Guo L., Wen P. (2008). Arrested proliferation and signal transduction of ERK5 in β-Glucan treated RAW264.7 macrophage cell line. J. Jiangsu Univ..

[24] Hao W., Zhao C., Li G., Wang H., Li T., Yan P., Wei S. (2023). Blue LED light induces cytotoxicity via ROS production and mitochondrial damage in bovine subcutaneous preadipocytes. Environ. Pollut..

[25] Roth G.A., Picece V., Ou B.S., Luo W., Pulendran B., Appel E.A. (2022). Designing spatial and temporal control of vaccine responses. Nat. Rev. Mater..

